# Editorial: Changing addiction problems and care responses during and after a major crisis: emergence of a ‘new normal'

**DOI:** 10.3389/fpubh.2024.1451141

**Published:** 2024-07-11

**Authors:** John Strang, Alexandra A. Deac, Harry Skipper, Julia RozanovaI

**Affiliations:** ^1^National Addiction Centre, Institute of Psychiatry, Psychology and Neuroscience, King's College London, London, United Kingdom; ^2^South London and Maudsley (SLaM) NHS Foundation Trust, London, United Kingdom; ^3^Department of Health Service and Population Research Department, King's College London, London, United Kingdom; ^4^Department of Psychological Medicine, King's College London, London, United Kingdom; ^5^AIDS Program, Yale University, New Haven, CT, United States

**Keywords:** crisis, addiction, substance use disorder, new normal, lessons

A major crisis can be understood as a significant societal event that disrupts the status quo in many spheres of life. Whether man-made (like revolutions, wars, or economic catastrophes) or natural disasters (like earthquakes, forest fires, or pandemics), crises are often polyphonic, with several disruptions happening simultaneously. Crises have occurred throughout the history of humanity, and the contemporary world continues to witness many, ranging from COVID-19 to wars to revolutions to natural disasters. Furthermore, crises put an enormous strain on societal resources and preparedness to mitigate their effects needs to be significantly improved and better understood. High-income countries may have relatively more resources prior to a crisis, but in the absence of crisis mitigation systems and processes, they may prove ill-prepared and still be left helpless. Lessons from Low- and Middle-Income Countries (LMIC) that have mitigated various risks over extended periods of time could thus be invaluable. This Research Topic brings together diverse research contributions on addiction in the context of crises, with a focus on both damage during and after a crisis, and the opportunities for innovation and improvement post-crisis. Collectively, these contributions begin to unpack key lessons about how addiction treatment systems at all levels—individual patients and providers, facilities, and institutions of national and international caliber—cope with and adapt to crises. They also examine the extent to which emergency solutions for providing addiction treatment services during a crisis are sustainable over time and the changes they can set in motion. Most importantly, this Research Topic shines light on empirical evidence that lifts the “fog of crisis”, to highlight the experiences and implications of crisis from the perspectives of different stakeholders and to build an understanding of the reality of what has happened.

This Research Topic features 13 articles and, while they all focus on substance use disorders including opioids (Kabembo) and alcohol (Nikitin et al.), they also capture the diversity of approaches to studying addiction problems and care responses during and after major crises in terms of methodologies, level of analysis, regional focus, and specific crises explored. Five articles report qualitative studies, three report findings from randomized controlled trials, four are based on survey research, and one reviews mixed-methods data from several sources. Of the 13 articles published in this Research Topic, six focus on HICs and seven on LMICs. However, all but one of the latter are written by HIC authors in collaboration with LMIC co-authors. This collaboration is undeniably positive but in our experience as Co-Editors who have encouraged and mentored many other LMIC authors (who sent in their ideas for a contribution), it was challenging for many potential contributors to set aside the demands of the crisis merely to complete and document academic analyses, largely because the crisis context required potential contributors in LMICs, like Mexico or Ukraine, to take on extra teaching, clinical care, and/or consultancy work to meet urgent response needs and to mitigate the escalating cost of living. Perhaps an immediate lesson to be learned is that research and timely analysis of crises need protected time through grant funding. Geographically, five articles focus on Ukraine, four on England, one on the United States, one on Zambia, one on Uganda, and one on the Western Europe region. Eleven articles investigate contemporary crises while one focuses on the aftermath of the Ugandan war a few years ago, and one article describes the status quo before COVID-19 and the war in Ukraine. From an editorial perspective, we would like to highlight three key themes in this corpus of articles.

The first key theme evident in all 13 articles is **the value of empirical research during and after the crisis**, to provide lessons for the future that are genuinely international (Sekeris et al.). Presenting evidence on how addiction treatment systems adapt to crises helps to overcome a mono-directional and inadvertently neo-colonial perspective, where practices from the West/HICs are often naively considered as universal therapeutic templates to be better rebuilt everywhere after a crisis. For example, articles by Nikitin et al. and Ponticello et al., which examine addiction treatment in Ukraine during the Russian invasion emphasize the importance of considering the impact of crises on the implementation process of OAT scale-up efforts and point to local Ukrainian-grown solutions for flexibility and responsiveness to patient needs in complex and rapidly changing environments. Similarly, Dellamura et al. take a step further and suggest that imposing on Ukrainian addiction treatment providers a system of accelerated performance indicators based on the recommendations of the Global Fund and other international authorities several years before the COVID-19 and war crises, without expanding the resources and addressing clinicians' concerns, increased the structural deficits in the healthcare system that the crisis exposed, while simultaneously fostering the ingenuity of healthcare workers to function in the absence of support, a crucial skill for survival under even more adverse circumstances. Describing the opioid epidemic as a man-made and avoidable crisis, McDonald et al. point to the role of transparency, accountability and the need for robust scientific research during crises.

The second thread running through all the articles is **the magnitude of the resilience of addiction care** in LMICs and HICs in the face of crisis, while also highlighting the limitations and costs of this resilience in terms of the burdens shouldered by individuals (patients and healthcare workers), families and caregivers, and facilities and care systems. As described in this Research Topic, across all contexts studied (from rural England to war-affected communities in Ukraine to post-conflict refugee camps in Uganda to an urban Californian community during the COVID-19 pandemic), addiction care was already, operating under conditions of overstretched resources pre-crisis (Makoha and Denov; Fstkchian et al.; Kabembo; Scott et al.). However, while the need for care intensified during the crisis and each context provided evidence of individual and collective ingenuity and adaptability that allowed, by and large, addiction care to be maintained for vulnerable patients, the uncomfortable question raised by the articles is the risk of working beyond capacity becoming solidified as the new normal. When the new normal is the crisis itself, then the context is permanently dangerous and unpredictable, and with the risk that resources will be permanently inadequate.

The final key theme to emerge from the Research Topic is **the value of local knowledge** (and the local experts who embody and practice it) as the principal ingredient **in crisis mitigation in addiction care**. No practice (e.g., allowing take-home doses of methadone for stable patients, or the use of telehealth) could prove universally sufficient without being promptly adapted and tailored to the local context, and articles by Galvez et al. and Ponticello et al. detail how this is being done during the war in Ukraine in prisons and in the community (Mazhnaya et al.). However, evidence from rural English communities during the COVID-19 crisis suggests that these practices need to be applied with caution (Scott et al.), both due to the relative digital illiteracy of some of the most vulnerable patients (Gilchrist et al.), and because the elimination of regular contact with the provider deprived such patients of the essential touch-base care points that gave them social support and medical advice (Campbell et al.).

In sum, we present this Research Topic as a valuable start to examining how addiction problems and care responses change during a major crisis and we call for continued work on this topic. First, we see extensive opportunities to integrate diverse evidence from different regions and across historical periods. More empirical and longitudinal research is needed to understand, for example, the responses to COVID-19 or the war in Ukraine (to take just two crises) over time, and to assess the long-term effects of any mitigation measures on individuals, facilities, and the national addiction care system. Systematic reviews of adaptations to addiction care during crises, as well as analyses of large international datasets, would be a crucial addition to the extant evidence base. Second, several article proposals that did not make it into this Research Topic have raised our awareness of gaps in knowledge that still need to be bridged. These concern geographic regions (e.g., Central Asia and Latin America) that remain unexplored in the current Research Topic, as well as substantive topics (e.g., drug trade/supply responses to crises; or the digital technologies in addiction care during and after crises) that still require attention. From conversations with potential authors, we realized the potential importance of articles that analyze the decision-making of local or (inter)national addiction care authorities during a crisis about how to re-organize and change patient care, in the absence of readily available guidelines for such decisions: these result in great uncertainty, multiple risks, and a heavy burden of responsibility if anything goes wrong, and this needs to be better understood, described and investigated. It is our hope that such research will be undertaken and featured in future Research Topics. Finally, we see this Research Topic as one of the first steps toward the genuine de-colonization of international guidelines for the provision of addiction care during crises. Evidence from different contexts needs to be examined and brought together to allow both HICs and LMICs to benefit from mutual learning.

It has been a privilege to guest edit this Research Topic and we thank all the contributors for their insightful research. We believe that this Research Topic can be useful to readers in academia, clinical care, public policy, and wherever there are work-related interests and/or lived and living experiences that involve addiction care, and we hope that this will provoke further reflection and discussion on what crises can teach us if we are willing to learn.

## Author contributions

JS: Conceptualization, Investigation, Methodology, Resources, Supervision, Writing – original draft, Writing – review & editing. AD: Conceptualization, Methodology, Project administration, Writing – original draft, Writing – review & editing. HS: Conceptualization, Investigation, Methodology, Project administration, Writing – original draft, Writing – review & editing. JR: Conceptualization, Methodology, Resources, Supervision, Writing – original draft, Writing – review & editing.

